# 3D region-growing nnU-Net improves pulmonary embolism detection on CTPA: a dual-cohort validation study

**DOI:** 10.1186/s41747-026-00693-3

**Published:** 2026-03-04

**Authors:** Ezio Lanza, Angela Ammirabile, Andrea Vanzulli, Costanza Lisi, Arosh Shavinda Perera, Ada Maria Antonella Lucia, Alessandra Mininni, Riccardo Levi, Marco Francone, Andrea Laghi

**Affiliations:** 1 Department of Biomedical Sciences, Humanitas University, Milan, Italy; 2 Department of Diagnostic and Interventional Radiology, IRCCS Humanitas Research Hospital, Milan, Italy; 3 Diagnostic and Interventional Radiology Residency Program, Università degli Studi di Milano, Milan, Italy; 4 Department of Diagnostic and Interventional Radiology, Fondazione IRCCS Istituto Nazionale dei Tumori, Milan, Italy; 5 Department of Radiological, Oncological and Pathological Sciences, Sapienza University of Rome, Rome, Italy

**Keywords:** Artificial intelligence, Computed tomography angiography, Deep learning, Major adverse cardiac events, Pulmonary embolism

## Abstract

**Objectives:**

We compared three customized nnU-Net models (A: baseline two-dimensional (2D); B: 2D + region-growing; C: three-dimensional (3D) + region-growing) for automated detection and blood clot volume (BCV) quantification of acute pulmonary embolism (PE) on computed tomography pulmonary angiography (CTPA), and to explore the association between BCV and clinical outcome.

**Materials and methods:**

We retrospectively screened 9,715 CTPA examinations (2015‒2024) to develop a dataset of 874 PE-positive and 339 PE-negative cases. A stratified subset (*n* = 437) with manually refined ground-truth segmentations was used for model training and internal validation. Region-growing in Models B and C included a 5-voxel negative buffer. Internal testing was performed on 776 cases (Humanitas dataset). External testing was performed on the public RSPECT-RSNA dataset. Performance metrics included accuracy, sensitivity, specificity, and area under the receiver operating characteristic curve (AUROC) at zero-clot and for optimized BCV threshold. Correlations between BCV, survival, and major adverse cardiovascular events (MACE) were analyzed.

**Results:**

Model C achieved the highest AUROC on external testing (0.868), outperforming Model A (0.843) and Model B (0.846). On internal testing at ROC-optimized threshold, Model C showed the highest accuracy (85.5%) and AUROC (0.909) compared to Model A (73.4%, 0.784) and Model B (76.0%, 0.816). Model C achieved 83.6% sensitivity and 79.5% accuracy at the zero-clot threshold on external data. BCV was not significantly associated with MACE or survival (*p* = 0.600).

**Conclusion:**

A locally trained 3D nnU-Net with region-growing demonstrated superior performance and generalizability on external data for automated PE detection on CTPA. However, BCV was not predictive of short-term clinical outcomes.

**Relevance statement:**

A locally developed nnU-Net models integrating volumetric 3D segmentation with region-growing offer robust, clinically acceptable performance for the detection of acute pulmonary embolism without the need for ROC-based thresholds.

**Key Points:**

- Our 3D nnU-Net model automates clot detection on CT scans in seconds and shows numerically higher performance than the 2D models.
- Built on local data, this framework enables institution-specific model training and validation to complement European conformity‒CE-marked tools and assess performance locally.
- High-sensitivity volumetric quantification reduces missed emboli, paving the way for personalized risk stratification and improved patient outcomes.

**Graphical Abstract:**

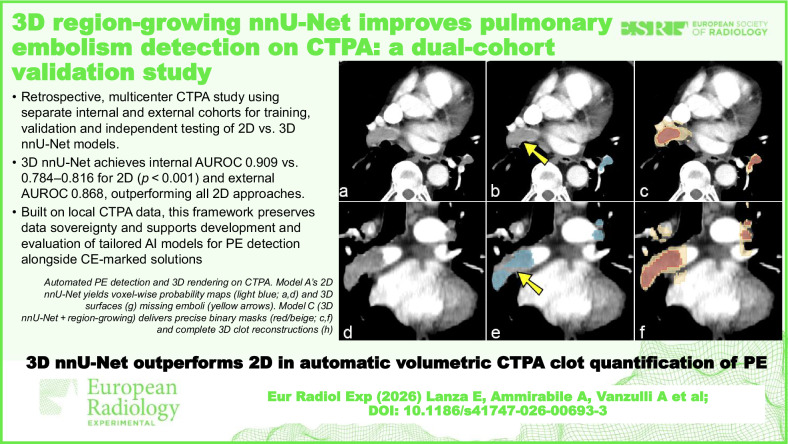

## Background

Pulmonary embolism (PE) is a leading cause of morbidity and mortality worldwide, typically resulting from thrombi obstructing pulmonary arteries, most often originating from deep vein thrombosis. The clinical manifestations of PE are highly variable, ranging from asymptomatic presentations to acute right ventricular failure and hemodynamic compromise [1], underscoring the critical importance of prompt and accurate diagnosis.

Computed tomography pulmonary angiography (CTPA) is the current standard of care for PE diagnosis [2, 3]. However, CTPA interpretation can be challenging due to the subtle imaging characteristics of pulmonary emboli, interobserver variability, and the potential for confounding anatomical structures, particularly for small or peripheral emboli [4–6]. These challenges highlight the need for robust, automated diagnostic tools that enhance consistency and reduce human error.

Recent advancements in artificial intelligence (AI) applied to medical imaging, specifically deep learning (DL) architectures for CT interpretation, have demonstrated promising potential to improve PE detection and quantification on CTPA [7–9].

Among DL frameworks, the nnU-Net architecture has become widely adopted for medical image segmentation due to its self-configuring architecture and strong performance across diverse tasks [10]. Prior studies have shown that nnU-Net can accurately identify PE, particularly central emboli [11, 12], and that automated quantification of blood clot volume (BCV) may serve as a valuable metric for objective PE severity assessment and clinical risk stratification [13–15]. However, the variable morphology of thrombi, their proximity to other vascular structures, and the potential for artifacts still pose challenges for automated detection, leading to potential false positives and negatives [16–18]. This variability necessitates the development of more sophisticated algorithms capable of reliably differentiating between thrombi and similar-appearing tissues. Therefore, there is growing interest in integrating spatially aware approaches, such as region-growing algorithms, with DL to refine embolus segmentation and potentially improve diagnostic accuracy. Yet, the specific value of these strategies in PE detection has not been systematically assessed.

This study aimed to address this gap by comparing the performance of three customized nnU-Net-based DL models for automated PE detection and BCV quantification on CTPA. Moreover, this research explores the relationship between automated BCV measurements and clinical outcomes, including survival and major adverse cardiovascular events (MACE).

## Materials and methods

### Study design and model development

**Fig. 1:**
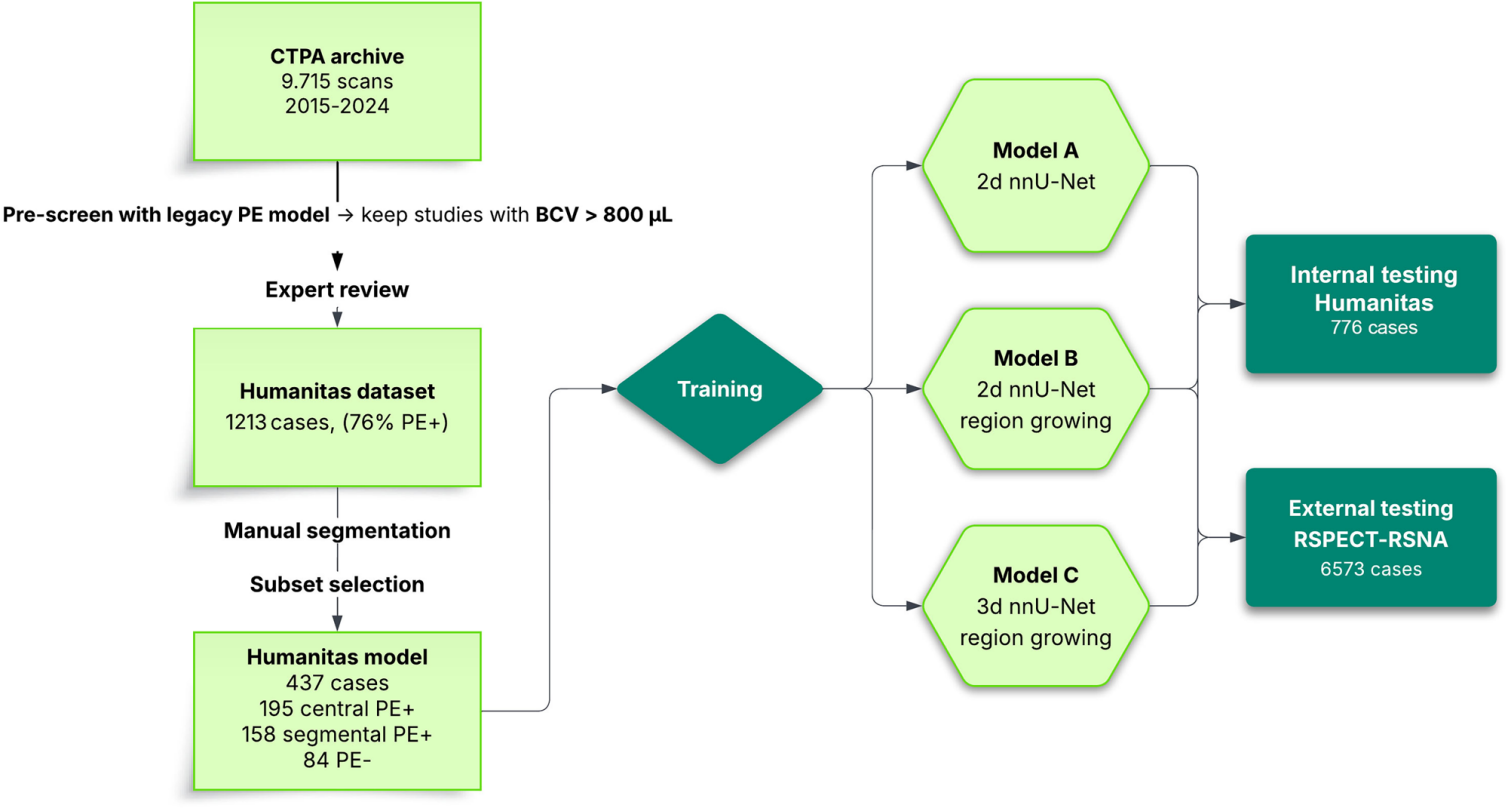
Study pipeline from CTPA screening through dataset curation, nnU-Net model training, and dual internal/external testing. PE, Pulmonary embolism, For RSPECT-RSNA dataset, see ref. [19]

This retrospective study was designed to develop and compare three distinct nnU-Net-based deep learning models for detecting and quantifying acute PE on CTPA. The models were developed and trained on a single-center dataset, with subsequent external testing on a large, publicly available dataset. All patient data was handled in compliance with institutional review board regulations and data privacy standards. Figure 1 illustrates the complete study workflow.

Three distinct models were created. Model A was a baseline two-dimensional (2D) nnU-Net. Model B was a 2D nnU-Net modified with a 5-voxel hard-negative border applied on each 2D slice; this was used to explicitly label voxels adjacent to thrombus as background and limit segmentation spillover along vessel walls. Model C was a full (three-dimensional) 3D nnU-Net implementing a 5-voxel isotropic hard-negative shell. This 3D margin was designed to encourage the network to learn the true 3D clot boundaries and to improve segmentation consistency across contiguous slices. The 5-voxel margin for Models B and C was chosen empirically to provide a sufficient buffer to disambiguate thrombus boundaries from adjacent tissues.

### Dataset curation and preparation

To assemble a local training dataset, we performed a retrospective screening of all CTPA examinations (*n* = 9,715) conducted at Humanitas Research Hospital between 2015 and 2024. All cases were fully anonymized upon retrieval. Inclusion criteria were adult patients who underwent contrast-enhanced CTPA acquired in the pulmonary arterial phase for suspected PE. Exclusion criteria were incomplete DICOM series, non-contrast scans, non-pulmonary-arterial phase studies, or severe technical artifacts precluding interpretation.

To identify positive cases, all eligible examinations were first screened using a previously published nnU-Net model [12] to technically assist case triage by flagging studies with a predicted clot volume > 800 µL. This tool served only as an enrichment step and did not determine ground truth; a proportion of flagged cases were false positives. All flagged scans, along with a sampled set of unflagged scans, were manually reviewed, and final PE-positive and PE-negative labels were assigned solely by expert interpretation. This process yielded a pool of 1,213 verified studies (874 PE-positive and 339 PE-negative). Crucially, diagnostic labels for this entire cohort were assigned exclusively by expert radiological interpretation, ensuring that every case, whether destined for training or independent testing, was manually verified.

From this verified pool, a subset of 437 studies was manually selected to create an optimized, data-rich development dataset. This selection was curated by expert radiological assessment to prioritize the clearest examples of central (*n* = 195) and peripheral (*n* = 158) PE, alongside high-quality negative cases (*n* = 84), thereby maximizing the anatomical distinctiveness of features provided to the model during training. Within the nnU-Net framework, this development set was automatically split 80:20 into a training set (*n* = 350) and an internal validation set (*n* = 87) used for hyperparameter tuning.

The remaining 776 verified studies were reserved as a held-out, independent internal test set. These cases were not used for training or tuning and served to evaluate model performance on local data independent of the development process (referred to as the independent Humanitas test dataset).

The ground truth for all PE-positive cases in the development set consisted of manual voxel-level thrombus segmentations. These were segmented and refined in 3D Slicer by a cardiothoracic radiologist with 8 years of experience; complex cases were further reviewed by a senior radiologist with over 15 years of experience to reach consensus. These manually curated segmentations served as the reference standard for training and for internal evaluation of the nnU-Net models. In contrast, the external dataset used the diagnostic labels provided by the original RSPECT-RSNA annotations.

### nnU-Net implementation details

All models were trained using the open-source nnU-Net framework [10], which automatically handles key preprocessing steps, including image resampling to a unified voxel spacing, intensity normalization, and data augmentation such as random rotations, scaling, and elastic deformations. The framework automatically configured the optimal patch size, batch size, and network architecture based on the dataset’s characteristics. Models were trained using a combination of Dice and cross-entropy loss on two workstations equipped with Nvidia RTX 4090 and RTX 3090 GPUs.

### Model evaluation

Two thresholding strategies were used: a zero-cutoff (0 mm³) to maximize sensitivity, and a model-specific, ROC-derived optimal cutoff to balance sensitivity and specificity.

For external testing, all three trained models were evaluated on a previously used [12] subset of the publicly available RSPECT-RSNA dataset [19], which comprised 6,573 CTPAs, of which 1,888 (29%) were positive for PE. The primary evaluation metrics included accuracy, sensitivity, specificity, the area under the receiver operating characteristic curve (AUROC), precision, recall, and false-positive rate. Performance was evaluated using two distinct PE classification strategies: an any-thrombus cutoff and an ROC-derived optimal cutoff. The first strategy, prioritizing maximum sensitivity, classified a scan as PE-positive if any thrombus volume was detected (BCV cutoff = 0 mm³). The second strategy applied a model-specific optimal BCV threshold to the external test set, determined using Youden’s J statistic on the internal validation set to balance sensitivity and specificity. Statistical analyses were performed using Python with the SciPy and scikit-learn libraries. 95% confidence intervals were calculated using bootstrapping (1,000 iterations). AUROC values were compared between models using the DeLong test, with statistical significance set at a two-sided *p*-value < 0.05 after Bonferroni correction for multiple comparisons.

### Clinical outcome analysis

To assess the clinical utility of automated BCV, a correlation analysis was performed between thrombus volume and key clinical outcomes. For the local patient cohort, data on survival, all-cause mortality, tumor presence (malignancy), and MACE were extracted from the institutional electronic health record. MACE was defined as a composite of events such as heart failure, ischemic heart disease, acute coronary syndromes, cardiac arrest, chest pain of suspected ischemic origin, and cardiac involvement related to systemic complications, as identified by International Classification of Diseases, Tenth Revision (ICD-10) codes occurring within 30 days following the index CTPA.

The primary statistical analysis evaluated the association between BCV (as a continuous and dichotomous variable using the 800 µL threshold) and MACE and survival time. Point-biserial correlation was used for binary outcomes (MACE, tumor presence), and Pearson correlation was used for survival time. Additionally, a post hoc analysis was performed to investigate the association between the presence of a known tumor and overall survival. All analyses were performed in Python, with statistical significance defined as a two-sided *p*-value < 0.05.

## Results

Baseline clinical characteristics of the internal cohort are summarized in Table 1. The median age was 71 years (interquartile range (IQR) = 20), 46.3% of patients had a known malignancy, and 10.1% experienced a major adverse cardiovascular event. The median survival time following the index CTPA was 387 days (IQR = 1,247). Outcome analyses were performed on this entire cohort.

**Table 1:** Internal cohort characteristics

Variable	Local dataset (*n* = 1,213)
Age (years), median [IQR]	71.0 [20.0]
Survival (months), median [IQR]	1,247.0 [387.0]
Tumor	46.3%
Cardiac events	7.0%
Stroke	4.5%
Major adverse cardiovascular events	10.1%
Pulmonary embolism positive	72.1%

*IQR* Interquartile range

### Model performance for PE detection

**Fig. 2:**
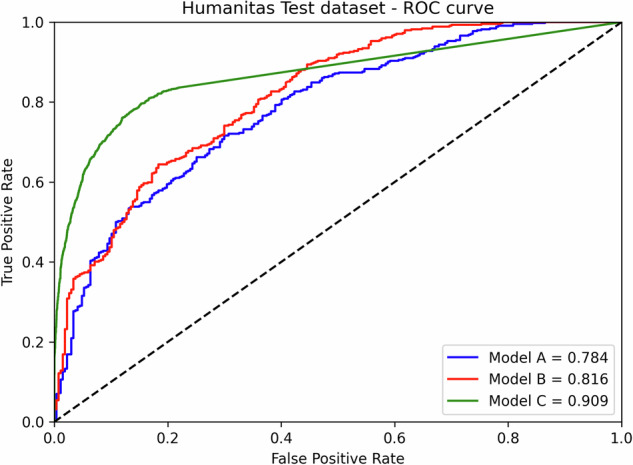
Receiver operating characteristics analysis displaying the accuracy of the three models at internal testing

**Fig. 3:**
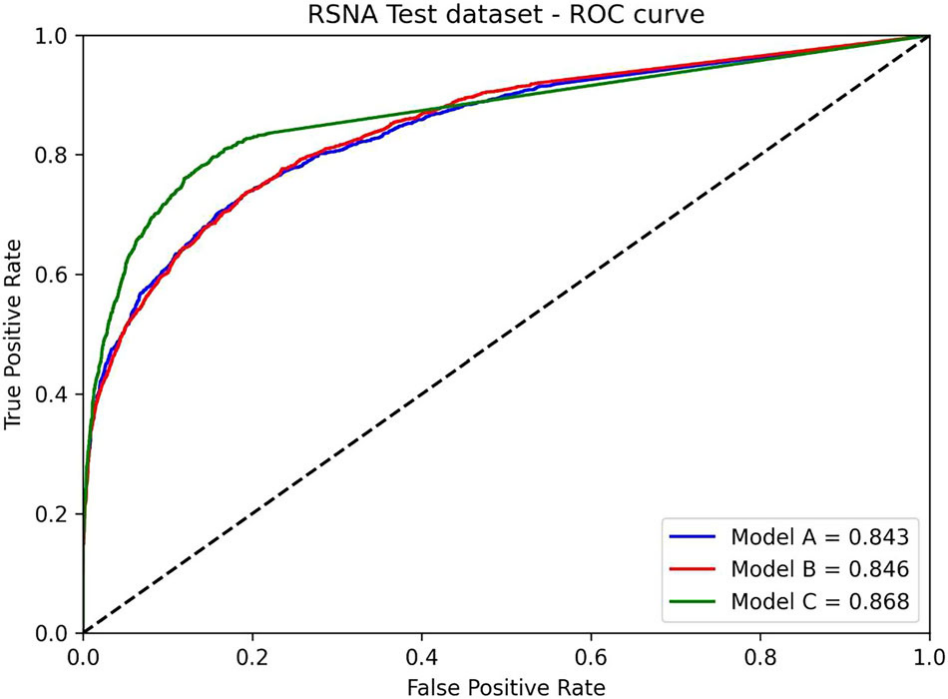
Receiver operating characteristics analysis displaying the accuracy of the three models at external testing

**Fig. 4:**
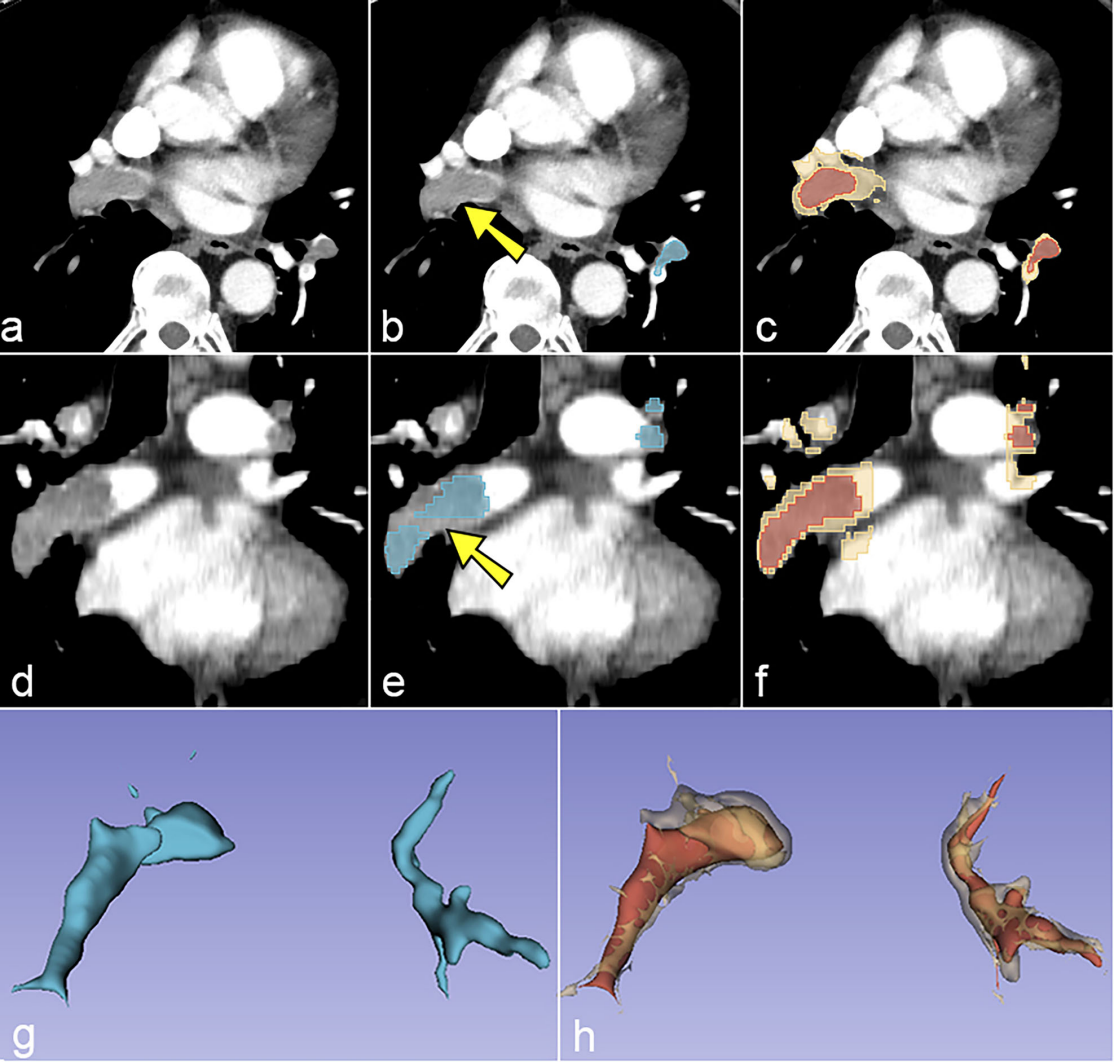
Automated detection and volumetric rendering of acute pulmonary embolism on CTPA in a single patient. **a**, **d** Original axial CTPA slices at two levels. **b**, **e** Model A (baseline 2D nnU-Net) voxel-wise probability map (light blue overlay) and (**g**) the corresponding 3D surface reconstruction of the clot. Yellow arrows indicate embolic foci missed by Model A but correctly identified by Model C. **c**, **f** Model C (3D nnU-Net + region-growing algorithm) final binary masks (red fill with beige outline) show more complete clot coverage on the same slices, along with (**h**) the matching 3D surface reconstruction of the clot. 2D, Two-dimensional; 3D, Three-dimensional; CTPA, Computed tomography pulmonary angiography

The diagnostic performance of the three nnU-Net models was evaluated using the internal (Humanitas) and external (RSPECT-RSNA) datasets. Overall, Model C (3D with region-growing) demonstrated the most robust performance, both in internal (Fig. 2) and external testing (Fig. 3). At its optimal cutoff of 1,075.6 mm³, Model C achieved the highest AUROC on external data (0.868) with an accuracy of 0.816, although the difference compared to Models A (0.843) and B (0.846) was not statistically significant (*p* = 0.601 and *p* = 0.895, DeLong test). A representative case comparing segmentation performance across models is shown in Fig. 4, and key trade-offs between the models and thresholding strategies are summarized in Table 2.

**Table 2:** Accuracy of the three different models in detecting and quantifying pulmonary embolism

Model A					
Dataset	Cutoff (mm^3^)	Accuracy	Sensitivity	Specificity	AUROC
Humanitas	0.000	0.627	1.000	0.008	0.784
RSPECT	0.000	0.578	0.918	0.443	0.843
Humanitas	1,317.662	0.734	0.865	0.517	0.784
RSPECT	1,317.662	0.806	0.342	0.990	0.843
Model B					
Humanitas	0	0.639	1.000	0.038	0.816
RSPECT	0	0.592	0.920	0.461	0.846
Humanitas	1,199.352	0.760	0.876	0.566	0.816
RSPECT	1,199.352	0.807	0.351	0.989	0.846
Model C					
Humanitas	0	0.753	0.998	0.345	0.909
RSPECT	0	0.795	0.836	0.779	0.868
Humanitas	1,075.623	0.855	0.903	0.775	0.909
RSPECT	1,075.623	0.816	0.382	0.989	0.868

*AUROC* Area under the receiver operating characteristic curve

When using the ROC-derived cutoffs on the external dataset, all models achieved high specificity (Model A: 0.990, Model B: 0.989, Model C: 0.989). However, this came at the cost of significantly lower sensitivity, with Model C yielding the highest sensitivity in this group at 0.382. In internal testing, Model B showed the best-balanced performance with the highest accuracy (0.760) and sensitivity (0.876) at its optimal cutoff. To simulate a high-sensitivity screening scenario, a 0 mm³ cutoff was applied. In the external testing, this approach substantially increased sensitivity for all models. Models A and C performed best, with identical sensitivity (0.836) and accuracy (0.795). Model B, while having the highest sensitivity in this scenario (0.920), had lower accuracy (0.592) due to a higher false-positive rate.

### Association between BCV, MACE, and survival

We evaluated the predictive ability of AI-quantified BCV for MACE and survival within our local patient cohort (Table 1). The cohort’s median age was 71 years (IQR = 20), and the median survival time after CTPA was 387 days (IQR = 1,247). Notably, 46.3% of patients had a known tumor diagnosis, and 10% experienced a MACE event.

The analysis revealed no statistically significant correlation between BCV and clinical outcomes. When analyzed as a continuous variable, BCV showed no association with MACE (point-biserial correlation *r* = -0.017, *p* = 0.600) or survival (*r* = -0.004, *p* = 0.910). Similarly, when BCV was dichotomized using the clinically relevant 800 µL threshold, a high clot burden (> 800 µL) did not correlate with either MACE (*r* = -0.027, *p* = 0.390) or survival (*r* = -0.018, *p* = 0.590).

Interestingly, although BCV was not predictive, a post hoc analysis showed a weak, statistically significant inverse association between the presence of a known tumor and patient survival (*r* = -0.258; *p* < 0.001), suggesting a possible prognostic impact of underlying malignancy in this cohort.

## Discussion

In this study, we compared three customized nnU-Net models for the automated detection and quantification of PE on CTPA. Our principal finding is that the three-dimensional model incorporating a region-growing algorithm (Model C) provided the most robust and generalizable performance. This model achieved the highest AUROC of 0.868 on the external dataset, reinforcing the hypothesis that full volumetric context is superior to 2D slice-based approaches for this complex segmentation task.

These results align with previous findings from Lanza et al [12], who also reported enhanced PE detection using a 3D nnU-Net, and with other studies [20–25] that have successfully used volumetric DL for precise embolus delineation. Liu et al, using U-Net DL segmentation, achieved notably high sensitivity (94.6%) and specificity (76.5%), demonstrating the effectiveness of volumetric segmentation in precisely delineating embolic material, especially in the central pulmonary arteries [22]. Consistently, Aydın et al reported comparable high sensitivity (95%) using similar DL volumetric segmentation techniques [23]. The advantage of the 3D approach appears to stem from its ability to process spatial information across slices, which is critical for differentiating true emboli from confounding structures such as vessel bifurcations, lymph nodes, and motion artifacts. Adding a hard-negative margin through our region-growing algorithm likely further refined this capability. This technique discourages over-segmentation by explicitly training the model to recognize the boundary between a thrombus and adjacent tissues. It promotes more precise predictions, a crucial factor when moving from simple detection to accurate volumetric quantification.

A key finding with direct clinical relevance is the trade-off between sensitivity and specificity governed by the BCV cutoff threshold. Applying a model-specific, ROC-derived cutoff resulted in excellent specificity (0.989 for Model C) at the cost of low sensitivity (0.382). While such a high-specificity tool could serve as a reliable “second reader” to confirm suspected cases, its low sensitivity limits its use for primary screening. Conversely, a zero-cutoff strategy, classifying any detected thrombus as positive, yielded a high sensitivity (0.836) and a clinically acceptable specificity (0.779) on external data. This performance profile is highly desirable in an emergency radiology setting, where maximizing detection to avoid missing a potentially fatal diagnosis is paramount. This inherent balance is a well-documented challenge in PE algorithm development, as noted by Huang et al [24] and Ayobi et al [26].

A significant limitation that must be carefully considered when interpreting our findings is the selection bias introduced during the curation of our local training dataset. Using a preliminary model and an 800 µL BCV threshold to screen for candidate cases, we inadvertently enriched our training set with cases of moderate-to-high thrombus burden. Consequently, the models were likely undertrained on the subtle imaging features of small, peripheral, or subsegmental emboli. This bias is a probable explanation for the notable drop in sensitivity observed on the external dataset, which can be assumed to contain a more natural and broader distribution of PE severity. This highlights a critical challenge in AI development: an enriched dataset may expedite initial training but can compromise the model’s ultimate real-world generalizability. Similarly, previously published external validation studies, such as those by Langius-Wiffen et al [27] and Talon et al [28], have reported significant performance declines when externally trained AI models are introduced into new clinical environments. Prospective multicenter validation studies would be particularly valuable in examining the impact of institutional fine-tuning and evaluating clinical outcomes associated with AI-assisted PE management.

Our evaluation compared only locally trained models and did not include a head-to-head comparison with CE-marked PE detection software. Commercial systems have demonstrated strong performance in prior studies, although reported accuracy varies across clinical environments [29]. Future work should include direct benchmarking against approved solutions in a prospective clinical workflow.

Our study also found no significant correlation between AI-quantified BCV and MACE or survival. This suggests that, in isolation, thrombus volume may be an insufficient biomarker for risk stratification in PE. The prognostic impact of a PE is likely multifactorial, dependent on the patient’s underlying cardiopulmonary reserve and the degree of resultant hemodynamic compromise, such as right ventricular strain. The moderate inverse correlation we observed between tumor presence and survival (*p* < 0.001) supports this view, indicating that, in our cohort, underlying comorbidities, such as malignancy, were possibly more determinants of outcome than the clot burden itself. Future risk-stratification models should therefore aim to integrate BCV with other automated imaging biomarkers, such as the right-to-left ventricular diameter ratio, and clinical data.

Despite these limitations, a key methodological strength of our work is the development and validation of an algorithm using local institutional data, without reliance on third-party commercial platforms. This strategy supports data privacy and institutional data sovereignty, providing a transparent and adaptable research framework aligned with evolving imaging protocols and patient characteristics. In the context of EU MDR regulations [30], this approach is not intended to suggest replacing CE-marked solutions; rather, it illustrates how local model development and validation can complement regulatory-approved tools by enabling institutions to assess performance using their own data and explore potential fine-tuning strategies where appropriate and legally permitted.

While previous work utilized the publicly available, large-scale RSPECT dataset for training, achieving notably high diagnostic performances [12], we explicitly chose an entirely local dataset. This decision was made to reflect real-life hospital scenarios and represents a practical and sustainable model for the clinical translation of AI.

In conclusion, this study demonstrates the performance of a 3D nnU-Net model with a region-growing algorithm for PE detection. It provides a research framework for developing and evaluating locally trained PE detection models and may inform future strategies for validating or fine-tuning CE-marked solutions in specific institutional settings, in compliance with EU MDR requirements [31]. While the presented algorithms require further optimization and prospective validation in real-world clinical workflows, they establish a strong foundation. Future work must focus on training with more heterogeneous datasets to build a comprehensive model that integrates volumetric, functional, and clinical data, moving beyond simple detection toward robust, automated patient risk stratification.

## Data Availability

The datasets generated and/or analyzed during the current study are available from the corresponding author on reasonable request.

## Abbreviations

2D: Two-dimensional
3D: Three-dimensional
AI: Artificial intelligence
AUROC: Area under the receiver operating characteristic curve
BCV: Blood clot volume
CTPA: Computed tomography pulmonary angiography
DL: Deep learning
IQR: Interquartile range
MACE: Major adverse cardiovascular events
PE: Pulmonary embolism
